# *YTHDF2* Gene rs3738067 A>G Polymorphism Decreases Neuroblastoma Risk in Chinese Children: Evidence From an Eight-Center Case-Control Study

**DOI:** 10.3389/fmed.2021.797195

**Published:** 2021-12-14

**Authors:** Huijuan Zeng, Meng Li, Jiabin Liu, Jinhong Zhu, Jiwen Cheng, Yong Li, Jiao Zhang, Zhonghua Yang, Li Li, Haixia Zhou, Suhong Li, Huimin Xia, Yan Zou, Jing He, Tianyou Yang

**Affiliations:** ^1^Department of Pediatric Surgery, Guangzhou Institute of Pediatrics, Guangdong Provincial Key Laboratory of Research in Structural Birth Defect Disease, Guangzhou Women and Children's Medical Center, Guangzhou Medical University, Guangzhou, China; ^2^Department of Clinical Laboratory, Biobank, Harbin Medical University Cancer Hospital, Harbin, China; ^3^Department of Pediatric Surgery, The Second Affiliated Hospital of Xi'an Jiaotong University, Xi'an, China; ^4^Department of Pediatric Surgery, Hunan Children's Hospital, Changsha, China; ^5^Department of Pediatric Surgery, The First Affiliated Hospital of Zhengzhou University, Zhengzhou, China; ^6^Department of Pediatric Surgery, Shengjing Hospital of China Medical University, Shenyang, China; ^7^Kunming Key Laboratory of Children Infection and Immunity, Yunnan Key Laboratory of Children's Major Disease Research, Yunnan Institute of Pediatrics Research, Yunnan Medical Center for Pediatric Diseases, Kunming Children's Hospital, Kunming, China; ^8^Department of Hematology, The Second Affiliated Hospital and Yuying Children's Hospital of Wenzhou Medical University, Wenzhou, China; ^9^Department of Pathology, Children Hospital and Women Health Center of Shanxi, Taiyuan, China

**Keywords:** neuroblastoma, *YTHDF2*, rs3738067, polymorphism, susceptibility

## Abstract

Neuroblastoma is a primary malignancy mainly occurring in children. We have reported that polymorphisms of several N6-methyladenosine (m6A) RNA modification-related genes contributed to neuroblastoma risk in previous studies. YTHDF2, a “reader” of RNA m6A modification, is involved in cancer progression. Here, we estimated the association between a *YTHDF2* gene rs3738067 A>G polymorphism and neuroblastoma susceptibility in 898 neuroblastoma patients and 1,734 healthy individuals from China. We found that the rs3738067 A>G could decrease neuroblastoma risk [AG vs. AA: adjusted odds ratio (OR) = 0.76, 95% confidence interval (CI) = 0.64–0.90, *P* = 0.002; AG/GG vs. AA: adjusted OR = 0.81, 95% CI = 0.69–0.95, *P* = 0.011). Besides, the rs3738067 AG/GG genotype was related to reduced neuroblastoma risk in the following subgroups: children aged 18 months and under, boys, patients with tumors originating from retroperitoneal, patients at clinical stage IV, and cases at clinical stages III plus IV. Importantly, false-positive report probability analysis proved our significant results worthy of close attention of. The expression quantitative trait locus analysis results revealed that the rs3738067 was associated with the expression of *YTHDF2*.

## Introduction

Neuroblastoma is a prevalent malignancy originating from precursor cells of the sympathetic nervous system, and it mainly affects infants and children under 5 years of age (1). Neuroblastoma with high aggressiveness often progresses quickly, leading to a disappointing prognosis and high recurrence rate. Although some patients experience mild or no treatment exhibit spontaneous regression (2), more than half of patients with high-risk neuroblastoma die even with multimodality treatment (3). Due to the complex nature of the disease, the pathogenesis of neuroblastoma is still far from clear. Increasing evidence suggests that the gradual accumulation of adverse genetic alterations leads to the transformation of normal cells to cancer cells (4). Therefore, it is essential to uncover the detrimental genetic changes in neuroblastoma to screen for high-risk individuals and explore potentially effective treatment.

In recent years, researchers have achieved dramatic advancements in the genetic etiology of neuroblastoma (5). Of note, genome-wide association studies (GWASs) have emerged as a powerful tool for exploring the causal genetic mechanisms of human diseases, including tumors (6). Chromosome instability was considered as one of the major causes in neuroblastoma oncogenesis (7). Two studies demonstrated that neuroblastoma shares common DNA variants with malignant cutaneous melanoma (8) and congenital heart disease (9). Currently, some single-nucleotide polymorphisms (SNPs) related to neuroblastoma susceptibility have been identified by GWASs and studies with candidate gene strategy, including *LMO1* (10, 11), *METTL14* (12), *PARP1* (13), *MTHFR* and *VDR* (14). Nevertheless, the genetic variations known presently are not sufficient to fulfill the genetic landscape in neuroblastoma.

N6-methyladenosine (m^6^A) is the most popular post-transcriptional modification of RNAs in eukaryotes, particularly in messenger RNAs (mRNAs) (15). RNA m^6^A modification is a dynamic and reversible process regulated by methyltransferases (known as writers) and demethylases (known as erasers). RNAs with m^6^A modifications can be recognized by some RNA binding proteins (named readers), which decide the different destinies of the modified RNA (16). As a member of the YTH domain family, YTHDF2 functions as an m^6^A reader to modulate the translation, location, and stability of targeted mRNA (17). Emerging evidence has suggested that dysregulated m^6^A modifications are tightly implicated in various diseases, especially cancers (18). Many studies have demonstrated the involvement of YTHDF2 in the regulation of m^6^A modified targets in cancer development (19). However, there are few reports about SNPs in the *YTHDF2* gene and tumor risk.

We carried out a multi-center epidemiology study among Chinese children to analyze the association between the SNPs in the key m6A modification modulator gene *YTHDF2* and neuroblastoma susceptibility.

## Materials and Methods

### Sample Selection

This work was conducted with the approval of the Institutional Review Board of Guangzhou Women and Children's Medical Center, with 898 neuroblastoma patients registered in eight hospitals (Guangzhou, Zhengzhou, Wenzhou, Xi'an, Taiyuan, Kunming, Changsha, Shenyang) in China and 1,734 age- and gender-matched healthy controls involved in previous studies (Supplementary Table S1) (20, 21). All participants have signed informed consent.

### Polymorphism Selection and Genotyping

Only one potential functional SNP in the *YTHDF2* gene (rs3738067 A>G) was chosen and genotyped in this study. Selection criteria and genotyping by TaqMan methodology were described previously (22, 23). The *YTHDF2* gene rs3738067 A>G is located in transcription factor binding sites (TFBS) and might affect transcription activity as predicted by SNPinfo (https://snpinfo.niehs.nih.gov/snpinfo/snpfunc.html).

### Statistical Analysis

The Chi-square test was applied to measure the compliance of alleles at individual loci in controls with the Hardy-Weinberg equilibrium (HWE) and the differences of selected demographic variables between patients and controls. Logistic regression analyses determined crude or adjusted odds ratios (ORs, adjusted for age and gender) with respective 95% confidence intervals (CIs) to analyze the association of *YTHDF2* gene polymorphism with neuroblastoma risk. False-positive report probability (FPRP) analysis was applied to estimate the deserving attention of *YTHDF2* gene polymorphism in neuroblastoma as described before (12). In brief, three parameters were used to determine FPRP values, including statistical power, *P*-value, and prior probability representing a real association between the SNP and disease. We set 0.2 as an FPRP threshold and assigned a prior probability of 0.1 to detect an OR of 1.5 (for risk effects) or 0.67 (for protective effects) for the association of genotypes with neuroblastoma susceptibility. The association of the rs3738067 A>G with *YTHDF2* expression was determined in the GTEx portal (https://www.gtexportal.org/home/) via eQTLs analysis. *P* < 0.05 was taken as statistically significant. Analyses were processed with SAS 9.1 (SAS Institute).

## Results

### Association of *YTHDF2* rs3738067 A>G With Neuroblastoma Risk

The genotyping of *YTHDF2* was successfully screened in 896 neuroblastoma patients and 1,733 controls. The genotype distribution of *YTHDF2* rs3738067 A>G polymorphism and its relation to neuroblastoma susceptibility was indicated in Table 1. The frequency of the *YTHDF2* rs3738067 A>G genotype coincided with HWE among the controls (HWE=0.359). The minor allele frequency (MAF) of *YTHDF2* rs3738067 A>G polymorphism, was 0.2591 for the controls and 0.2410 for the cases. Based on the results of the single-locus analysis, we found that the G carriers of the rs3738067 were associated with decreased neuroblastoma risk (AG vs. AA: adjusted OR = 0.76, 95% CI = 0.64–0.90, *P* = 0.002; AG/GG vs. AA: adjusted OR = 0.81, 95% CI = 0.69–0.95, *P* = 0.011).

**Table 1:** *YTHDF2* rs3738067 A>G polymorphism and neuroblastoma susceptibility.

**Genotype**	**Cases (%) (*N* = 896)**	**Controls (%) (*N* = 1,733)**	***P* ^a^**	**Crude OR (95% CI)**	***P***	**Adjusted OR (95% CI)^b^**	***P* ^b^**
rs3738067 (HWE=0.359)							
AA	535 (59.71)	944 (54.47)		1.00		1.00	
AG	292 (32.59)	680 (39.24)		**0.76 (0.64–0.90)**	**0.002**	**0.76 (0.64–0.90)**	**0.002**
GG	69 (7.70)	109 (6.29)		1.12 (0.81–1.54)	0.498	1.12 (0.82–1.55)	0.478
Additive			0.134	0.91 (0.79–1.03)	0.134	0.91 (0.80–1.03)	0.143
Dominant	361 (40.29)	789 (45.53)	0.010	**0.81 (0.69–0.95)**	**0.010**	**0.81 (0.69–0.95)**	**0.011**
Recessive	827 (92.30)	1,624 (93.71)	0.172	1.24 (0.91–1.70)	0.173	1.25 (0.91–1.71)	0.165

*OR, odds ratio; CI, confidence interval; HWE, Hardy-Weinberg equilibrium*.

*Values were in bold if the P-values <0.05 or the 95% CIs excluded 1*.

a *χ^2^ test for genotype distributions between neuroblastoma cases and cancer-free controls*.

b *Adjusted for age and gender*.

### Stratification Analysis

After that, we assessed the relation between *YTHDF2* gene polymorphism and neuroblastoma susceptibility in subgroups classified via age, gender, sites of origins as well as clinical stages. As presented in Table 2, we detected that the rs3738067 AG/GG genotype carriers were linked to reduced neuroblastoma risk in subgroups of children with the age of 18 months and under (adjusted OR = 0.76, 95% CI = 0.58–0.98, *P* = 0.036), males (adjusted OR = 0.75, 95% CI = 0.60–0.93, *P* = 0.009), patients with retroperitoneal tumors (adjusted OR = 0.69, 95% CI = 0.54–0.88, *P* = 0.003), patients at clinical stage IV (adjusted OR = 0.68, 95% CI = 0.51–0.91, *P* = 0.009) and those at clinical stages III+IV (adjusted OR = 0.74, 95% CI = 0.59–0.93, *P* = 0.009).

**Table 2:** Stratify analysis for *YTHDF2* rs3738067 A>G polymorphism and neuroblastoma susceptibility.

**Variables**	**rs3738067 (cases/controls)**		**OR (95% CI)**	***P***	**AOR (95% CI)^a^**	***P* ^a^**
	**AA**	**AG/GG**				
Age, month						
≤ 18	204/373	140/340	**0.75 (0.58–0.98)**	**0.033**	**0.76 (0.58–0.98)**	**0.036**
>18	331/571	221/449	0.85 (0.69–1.05)	0.128	0.86 (0.70–1.06)	0.158
Gender						
Females	238/415	168/329	0.89 (0.70–1.14)	0.353	0.90 (0.71–1.15)	0.411
Males	297/529	193/460	**0.75 (0.60–0.93)**	**0.010**	**0.75 (0.60–0.93)**	**0.009**
Sites of origin						
Adrenal gland	141/944	107/789	0.91 (0.69–1.19)	0.481	0.91 (0.69–1.19)	0.477
Retroperitoneal	202/944	116/789	**0.69 (0.54–0.88)**	**0.003**	**0.69 (0.54–0.88)**	**0.003**
Mediastinum	121/944	92/789	0.91 (0.68–1.21)	0.518	0.92 (0.69–1.22)	0.552
Others	67/944	38/789	0.68 (0.45–1.02)	0.063	0.68 (0.45–1.03)	0.066
Clinical stages						
I	179/944	130/789	0.87 (0.68–1.11)	0.261	0.87 (0.68–1.12)	0.275
II	91/944	69/789	0.91 (0.65–1.26)	0.559	0.92 (0.66–1.27)	0.601
III	98/944	65/789	0.79 (0.57–1.10)	0.166	0.80 (0.58–1.11)	0.186
IV	147/944	84/789	**0.68 (0.52–0.91)**	**0.009**	**0.68 (0.51–0.91)**	**0.009**
4 s	14/944	4/789	0.34 (0.11–1.04)	0.059	0.37 (0.12–1.15)	0.085
I+II+4 s	270/944	199/789	0.88 (0.72–1.08)	0.232	0.88 (0.72–1.09)	0.239
III+IV	245/944	149/789	**0.73 (0.58–0.91)**	**0.006**	**0.74 (0.59–0.93)**	**0.009**

*OR, odds ratio; CI, confidence interval; AOR, adjusted odds ratio*.

*Values were in bold if the P-values <0.05 or the 95% CIs excluded 1*.

a *Adjusted for age and gender, omitting the corresponding stratify factor*.

### FPRP Analysis

An FPRP analysis was implemented to verify whether our significant findings deserve attentions. As shown in Table 3, the significant association for rs3738067 A>G (AG vs. AA, AG/GG vs. AA, males, retroperitoneal, clinical stage IV, and III+IV) remained noteworthy at the prior probability level of 0.1.

**Table 3:** False-positive report probability analysis for significant findings for the association between *YTHDF2* rs3738067 A>G polymorphism and neuroblastoma susceptibility.

**Genotype**	**Crude OR(95% CI)**	***P* ^a^**	**Statistical power^b^**	**Prior probability**				
				**0.25**	**0.1**	**0.01**	**0.001**	**0.0001**
AG vs. AA	0.76 (0.64–0.90)	0.002	0.929	**0.005**	**0.016**	**0.153**	0.646	0.948
AG/GG vs. AA	0.81 (0.69–0.95)	0.010	0.986	**0.030**	**0.086**	0.508	0.913	0.991
≤ 18 months	0.75 (0.58–0.98)	0.033	0.808	**0.108**	0.267	0.800	0.976	0.998
Males	0.75 (0.60–0.93)	0.010	0.833	**0.033**	**0.093**	0.530	0.919	0.991
Retroperitoneal	0.69 (0.54–0.88)	0.003	0.582	**0.015**	**0.043**	0.330	0.833	0.980
Stage IV	0.68 (0.52–0.91)	0.009	0.561	**0.045**	**0.124**	0.608	0.940	0.994
Stage III+IV	0.73 (0.58–0.91)	0.006	0.765	**0.021**	**0.061**	0.416	0.878	0.986

*OR, odds ratio; CI, confidence interval*.

*The results were in bold if the false-positive report probability <0.200*.

a *Chi-square test was used to calculate the genotype frequency distributions*.

b *Statistical power was calculated using the number of observations in the subgroup and the OR and P-values in this table*.

### Effect of rs3738067 A>G on the Expression of *YTHDF2*

**Figure 1:**
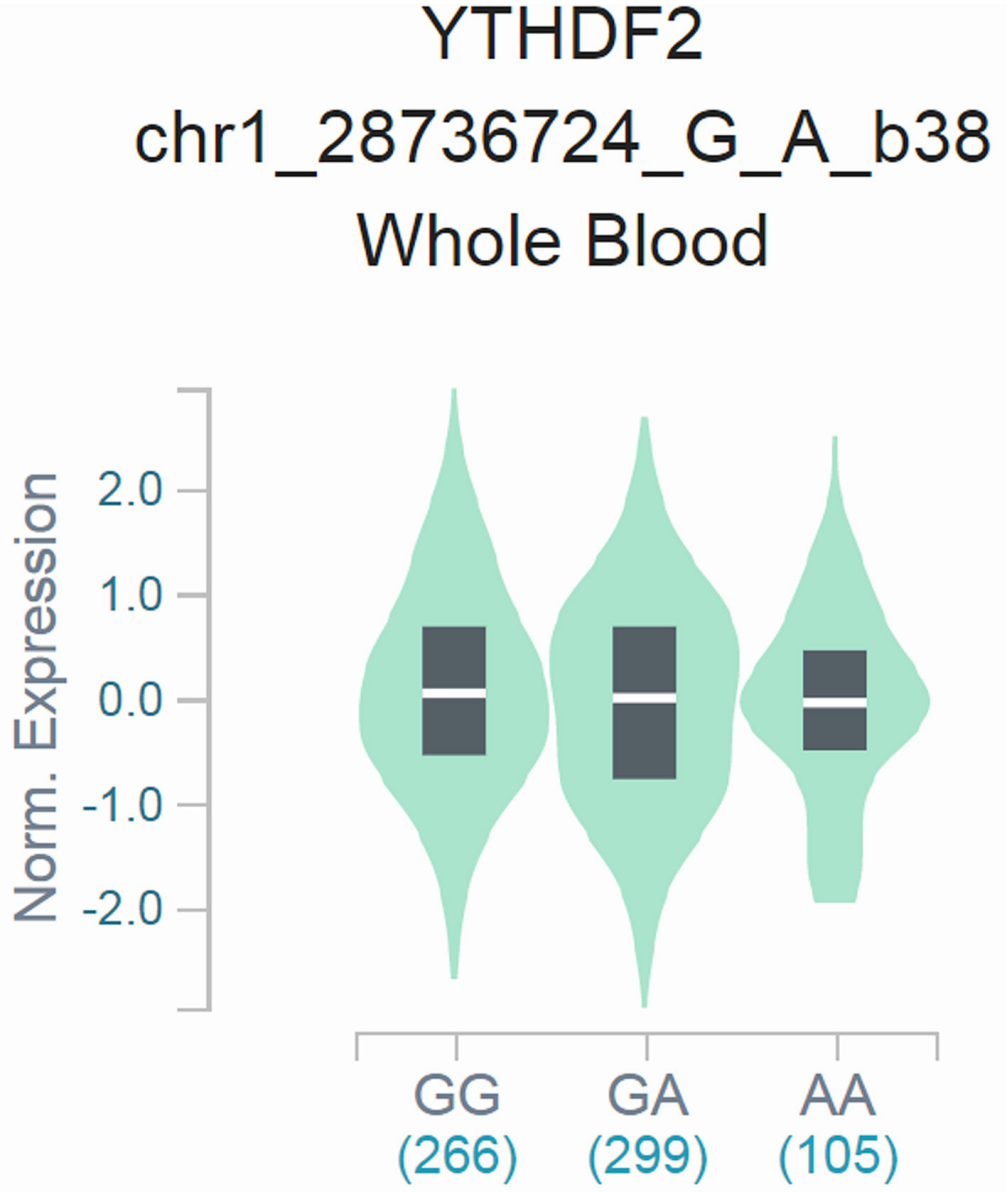
Functional relevance of rs3738067 A>G to *YTHDF2* expression in GTEx database. The rs3738067 A allele had a significant association with *YTHDF2* level alteration in the whole blood [*P*=1.9*10^−5^, normalized effect size (NES) = 0.084].

To confirm the functional relevance of rs3738067 A>G to the mRNA expression of *YTHDF2*, Cis-expression quantitative trait loci (eQTLs) analysis of the rs3738067 A>G and *YTHDF2* expression was estimated using GTEx data. Results manifested that the rs3738067 A allele was related to increased *YTHDF2* expression levels in the whole blood [Figure 1, *P*=1.9^*^10^−5^, normalized effect size (NES)=0.084].

## Discussion

Though many genetic variants linking to neuroblastoma susceptibility have been recognized, further efforts are needed to fully understand this disease's genetic landscape. The present work verified the *YTHDF2* rs3738067 A>G could reduce neuroblastoma risk for the first time. YTHDF2 is an m^6^A modification “reader” which recognizes m^6^A-modified mRNAs to modulate the translation and stability of targeted mRNA (24). The roles of *YTHDF2* in tumors are critical but controversial. For instance, As shown by Zhong et al., YTHDF2 restrained tumor cell growth in hepatocellular carcinoma (25). Shen et al. reported that YTHDF2 repressed cell growth in gastric cancer through modulating FOXC2 expression (26). In contrast, Li and colleagues disclosed that YTHDF2 promoted cell proliferation and migration in ovarian cancer (27). However, the role of *YTHDF2* in neuroblastoma remains largely unknown.

Increasing evidence has indicated that genetic variations, including SNPs in m^6^A modification modulators, correlate closely with cancer progression (28). Also, a report has pointed that *YTHDF2* rs3738067 A>G polymorphism exhibits a significant inverse association with glioma risk (29). Our study evaluated the association of the *YTHDF2* gene SNP (rs3738067 A>G) with neuroblastoma susceptibility. The results showed that rs3738067 AG/GG genotype was related to reduced neuroblastoma risk in several subgroups, including children aged 18 months and under, males, patients with tumors originating from retroperitoneal, patients in clinical stages IV, and patients in clinical stages III+IV. We also performed FPRP tests to confirm if the obtained associations were noteworthy or not to provide further evidence of the reliability of our results. The association of the rs3738067 A>G with *YTHDF2* expression was determined in the GTEx portal via eQTLs analysis. The integrative analyses of eQTL and SNP information may provide more understanding about the complex disease-modulating network (30). However, further studies are needed to substantiate the association between the rs3738067 A>G polymorphism and mRNA expression levels of *YTHDF2*.

There are several limitations to this study. First, only one SNP (rs3738067 A>G) in the *YTHDF2* gene was evaluated. More studies will be performed to find other potential functional SNPs in the *YTHDF2* gene. Second, only Chinese children were involved in this study. Thus, the results of this study may not be applicable to other ethnic groups. Moreover, as neuroblastoma is a multifactorial tumor, only genetic analysis is not enough to estimate neuroblastoma risk, and this study failed to incorporate environmental and genetic-environmental factors.

Our work for the first time verified the significant correlation of *YTHDF2* gene rs3738067 A>G polymorphism with neuroblastoma risk, and this polymorphism is an intriguing locus for in-depth researches. However, the underlying biological mechanisms remain to be explored.

## Data Availability Statement

The original contributions presented in the study are included in the article/Supplementary Material, further inquiries can be directed to the corresponding author/s.

## Ethics Statement

The studies involving human participants were reviewed and approved by Institutional Review Board of Guangzhou Women and Children's Medical Center. Written informed consent to participate in this study was provided by the participants' legal guardian/next of kin.

## Author Contributions

HZe, TY, and JH contributed to conception and design of the study. ML and JL organized the original data. JZhu, JC, YL, JZha, ZY, LL, HZh, and SL provided the clinical tissue and blood samples for the study. HX and YZ provided some technical guidance. All authors contributed to the article and approved the submitted version.

## Funding

This study was supported by grants from the National Natural Science Foundation of China (No. 82173593), Natural Science Foundation of Guangdong Province (No. 2019A1515010360), and the Major Science and Technology Special Project of Wenzhou (No. ZY2020021). The Natural science foundation of Guangdong Province (No. 2020A1515011569). Guangzhou Science and Technology Innovation Commission (No. 201607010395). Guangzhou Health science and Technology Project (No. 20201A010018). Guangzhou Health science and Technology Project (No. 20211A011033).

## Conflict of Interest

The authors declare that the research was conducted in the absence of any commercial or financial relationships that could be construed as a potential conflict of interest.

## Publisher's Note

All claims expressed in this article are solely those of the authors and do not necessarily represent those of their affiliated organizations, or those of the publisher, the editors and the reviewers. Any product that may be evaluated in this article, or claim that may be made by its manufacturer, is not guaranteed or endorsed by the publisher.
