# Supplementary material for: YTHDF2 Gene rs3738067 A>G Polymorphism Decreases Neuroblastoma Risk in Chinese Children: Evidence From an Eight-Center Case-Control Study
Source: Front Med (Lausanne). 2021 Dec 14;8:797195. doi: 10.3389/fmed.2021.797195 (PMC8712649; doi:10.3389/fmed.2021.797195)
Supplement: Supplementary file 1 [file Table_1.DOC]

| **Table S1**. Frequency distribution of selected characteristics in neuroblastoma cases and cancer-free controls from eight hospitals | | | | | |
| --- | --- | --- | --- | --- | --- |
| Variables | Combined subjects (8 Centers) | | | | |
|  | Cases (n=898) | | Controls (n=1734) | | *P* ^a^ |
|  | No. | % | No. | % |  |
| Age range, month | 0.00-176.00 | | 0.004-156.00 | | 0.155 |
| Mean ± SD | 33.11±28.07 | | 30.41±24.90 | |  |
| ≤18 | 344 | 38.31 | 714 | 41.18 |  |
| >18 | 554 | 61.69 | 1020 | 58.82 |  |
| Gender |  |  |  |  | 0.236 |
| Female | 407 | 45.32 | 744 | 42.91 |  |
| Male | 491 | 54.68 | 990 | 57.09 |  |
| INSS stages |  |  |  |  |  |
| I | 310 | 34.52 | / | / |  |
| II | 160 | 17.82 | / | / |  |
| III | 163 | 18.15 | / | / |  |
| IV | 231 | 25.72 | / | / |  |
| 4s | 18 | 2.00 | / | / |  |
| NA | 16 | 1.78 | / | / |  |
| Sites of origin |  |  |  |  |  |
| Adrenal gland | 248 | 27.62 | / | / |  |
| Retroperitoneal region | 319 | 35.52 | / | / |  |
| Mediastinum | 214 | 23.83 | / | / |  |
| Other region | 105 | 11.69 | / | / |  |
| NA | 12 | 1.34 | / | / |  |
| SD, standard deviation.  ^a^ Two-sided χ^2^ test for distributions between neuroblastoma cases and cancer-free controls. | | | | | |
